# Identification of Heterosis-Associated Stable QTLs for Ear-Weight-Related Traits in an Elite Maize Hybrid Zhengdan 958 by Design III

**DOI:** 10.3389/fpls.2017.00561

**Published:** 2017-04-19

**Authors:** Hongjian Li, Qingsong Yang, Lulu Gao, Ming Zhang, Zhongfu Ni, Yirong Zhang

**Affiliations:** ^1^State Key Laboratory for Agrobiotechnology and Key Laboratory of Crop Heterosis Utilization (MOE), China Agricultural UniversityBeijing, China; ^2^National Maize Improvement Center of China, China Agricultural UniversityBeijing, China

**Keywords:** heterosis, QTL, Zhengdan 958, Design III, maize

## Abstract

Heterosis plays a decisive role in maize production worldwide, but its genetic basis remains unclear. In this study, we explored heterosis for ear-weight (EW)-related traits using a North Carolina Experiment III design (Design III) population derived from the elite maize hybrid Zhengdan 958. Quantitative trait loci (QTL) analysis was conducted based on phenotypic data collected from five environments using a high-density linkage map that consisted of 905 single nucleotide polymorphisms (SNP). A total of 38 environmentally stable QTLs were detected, and the numbers for the Z_1_ and Z_2_ populations were 18 and 20, respectively. All environmentally stable QTLs for Z_2_ were characterized by the overdominance effect (OD), which indicated that overdominance was one of the most important contributors to the heterosis of EW-related traits. Consistent with the significant positive correlations between EW-related traits, 9 genomic regions with overlapped QTLs for different traits were found and were located on chromosomes 1 (1), 3 (2), 4 (3), 7 (1), 8 (1), and 9 (1). Compared to previous reports, we found that the genomic regions for heterosis were not always congruent between different hybrids, which suggested that the combination of heterotic loci in different hybrids was genotype-dependent. Collectively, these data provided further evidence that the potential utilization of QTLs for heterosis may be feasible by pyramiding if we treat the QTLs as inherited units.

## Introduction

Heterosis or hybrid vigor, which was denoted as the high-ranking performance of the F_1_ hybrid relative to their parents, has played a decisive role in crop production for almost a century (East, 1908; Shull, 1908). However, constant efforts from different researchers have not led to consistent conclusions about the genetic mechanism of heterosis (Stuber, 1994; Hua et al., 2003; Radoev et al., 2008; Liang et al., 2015), which has hindered its use in crop improvement programs. To date, three main hypotheses have been proposed to explain the genetic basis of heterosis, including dominance and overdominance based on individual gene loci as well as epistasis based on the interactions among the genes. The dominance hypothesis emphasized the complementation of slightly deleterious recessive alleles that lie in the inbred parents (Jones, 1917). The overdominance hypothesis indicated that the performance of heterozygosity was superior to that of the homozygous condition (Shull, 1908; Hull, 1945). Epistasis attributed heterosis to interactions of genes from different loci (Richey, 1942; Powers, 1944).

Over the past two decades, quantitative genetics and the advance of molecular markers have provided powerful tools for the genetic analysis of heterosis in crops (Edwards et al., 1992). For example, Hua et al. (2003) found 33 single heterotic loci (HL) and that dominance by dominance interactions could adequately explain the genetic basis of heterosis in an elite rice hybrid using an “immortalized F_2_”. In contrast, in a reanalysis of their dataset with an ultra-high density SNP bin map, Zhou et al. (2012) concluded that overdominance/pseudo-overdominance was very important to heterosis of yield and that dominance by dominance interactions is important for heterosis of grain weight and tillers per plant in rice. Recently, Huang et al. (2016) found support for the partial dominance of heterozygous locus for yield-related traits and better-parent heterosis for overall performance by conducting a GWAS analysis of 10,074 F_2_ lines from 17 representative hybrid rice crosses. In rapeseed, epistasis along with all levels of dominance (partial dominance, dominance, and overdominance) were declared to be responsible for the expression of heterosis (Radoev et al., 2008; Li et al., 2012).

To date, several QTLs (genes) related to heterosis were fine mapped and/or cloned in various species. Using near isogenic lines (NILs), He et al. (2006) narrowed the yield-improving QTL *qGY2–1* down to a 102.9-kb region on rice chromosome 2 and found allelic expression variation in this gene cluster. Krieger et al. (2010) reported a gene named *SINGLE FLOWER TRUSS* (*SFT*) in tomato, which was the first example of a single overdominant gene for yield. Heterozygosity for loss-of-function alleles of *SFT* increases the yield by up to 60%. Xue et al. (2008) isolated a quantitative trait locus *Ghd7* from an elite rice hybrid, and this locus encoded a CCT domain protein. Analysis of backcross (BC) populations revealed that the dominance effect (D) was observed for the number of grains per panicle, plant height, and heading date because heterozygotes were close to the performance of the higher parent. In palm, Singh et al. (2013) cloned a *SHELL* gene that is a homolog of the MADS-box gene *SEEDSTICK*. This gene can improve the yield of mesocarp oil via heterodimerization, which provided a genetic explanation for single gene heterosis.

Maize has been applied as a model species for a long time in exploring heterosis for its high heterosis degree (Jones, 1922; Duvick, 2001). Grain yield and yield-related traits in maize were analyzed in various experimental designs. Adopting Design III, Stuber et al. (1992) did pioneering work to clarify the heterosis of grain yields and plant height with the aid of molecular markers using separate backcrosses. Their results showed that overdominance (or pseudo-overdominance) plays an especially important role in the phenomenon of heterosis. However, Cockerham and Zeng (1996) obtained a different conclusion for an alternative method of data analysis using the same data mentioned above, which considered dominance along with epistatic effects between linked QTLs to be the principal elements of heterosis in maize. The dominance hypothesis of grain yield and its related traits were also supported by the results of Tang et al. (2010), using an “IF_2_” design in an elite maize hybrid. Jiang et al. (2015) also concluded that dominance and epistatic QTLs are important for the heterosis of kernel-shape-related traits using a triple testcross population in maize. The ever-changing clues for heterosis in previous studies indicated that the genetic basis of heterosis is intricate and that further studies should be pursued.

The hybrid Zhengdan 958 is one of the most widely grown commercial varieties in China currently (~500 million hectares between 2001 and 2015) for its high planting density, good stress-tolerance, and high but stable yield (Li et al., 2009; Lai et al., 2010). To date, only one study based on F_2:3_ families derived from Zhengdan 958 was reported (Guo et al., 2011), but no research focusing on the genetic basis of heterosis underling Zhengdan 958 was found in the literature. The main objectives of this study were (1) to assess the level of heterosis for EW and its components; (2) to detect the QTLs and evaluate the effects related to heterosis; (3) to examine the role of epistasis in heterosis; and (4) to compare heterotic QTLs with previous studies.

## Materials and methods

### Plant materials

The high-heterosis hybrid Zhengdan 958 has been planted all over China. Its parental inbreeds belong to two different heterotic groups: Zheng 58 belongs to the PA heterotic group, a subgroup of SS, and Chang 7–2 belongs to TSPT heterotic group, a subgroup of NSS (Bai et al., 2015; Ma et al., 2016). Following the testcross (TC) progeny production scheme (Comstock and Robinson, 1952), 174 RILs (F_7_) derived from the hybrid Zhengdan 958 were used as pollen parents to cross the parental lines Zheng 58 [TC (Zheng 58)] and Chang 7–2 [TC (Chang 7–2)]. Due to a seed shortage in a few crosses, 162 RILs and their corresponding testcrosses (TCs) were employed.

### Field experiments

The two populations of TC progeny along with other materials (i.e., the parental lines, Zhengdan 958 and RILs) were field-tested in 2012 and 2013 on the experimental farm of Jilin Academy of Agricultural Sciences (Jilin province, China) and the experimental farm of the Xinjiang Academy of Agricultural Sciences (Xinjiang province, China) and, in 2012, on the Agronomy Farm of Jinghai (Tianjin, China) (Figure S1 and Table S1). The TCs were planted in a randomized complete block design with three replications at each location. Each plot included rows that were 4 m long with 0.67 m of space between rows. The population density was 45,000 plants per hectare. The 162 RILs, hybrid and the two parental lines of Zhengdan 958 were also planted using the same experimental design, which was near the TC experiment. All fields were well-watered with broad irrigation and rainfall. Other field management policies followed local standard practices.

Ten ears from consecutive plants in each plot were harvested and air-dried after maturity. Data for the following traits were collected: ear row number (ERN), ear diameter (ED), number of seeds per row (RSN), ear length (EL), one hundred seed weight (HSW), ear seed number (ESN), ear seed weight (ESW), and ear weight (EW).

### Data analysis

The percentage of heterosis was analyzed in the basic generations as mid-parent heterosis (MPH), which was computed as MPH = (F_1_-MP)/MP × 100, where MP represented the mid-parent value. Following the methods reported by Comstock and Robinson (1952) and Melchinger et al. (2007), the crosses of RILs to their parental lines Zheng 58 and Chang 7–2 were indicated as L_1i_ and L_2i_ (i = 1~162), respectively. The linear transformations were Z_1i_ = (L_1i_+L_2i_)/2 and Z_2i_ = L_2i_-L_1i_. A combined ANOVA over five environments was calculated to estimate variance components. Additive Variances (*V*_*A*_) within Z_1_ and dominance variances (*V*_*D*_) within Z_2_ were used to estimate the average degree of dominance D^*^ as (*V*_*D*_/*2V*_*A*_) ^0.5^, which stood for the degree of dominance over all separating loci (Cockerham and Zeng, 1996; Frascaroli et al., 2007; Melchinger et al., 2007).

The adjusted mean (Best Linear Unbiased Prediction, BLUP) values across five environments were calculated with the PROC MIXED procedure in SAS (SAS Institute Inc., North Carolina, USA). Broad-sense heritability ($h_{B}^{2}$) were calculated as $h_{B}^{2}$ = $\sigma_{g}^{2}$*/(*$\sigma_{g}^{2}+\sigma_{ge}^{2}$*/n*+σ^2^*/nr)*, where $\sigma_{g}^{2}$ is the genetic variance, $\sigma_{ge}^{2}$ is the genotype by environment interaction variance, σ^2^ is the error variance, *n* is the number of environments, and *r* is the number of replications of each experiment (Knapp et al., 1985; Churchill and Doerge, 1994). Correlation coefficients among traits were estimated using adjusted mean values for both Z_1_ and Z_2_.

### Genotyping and linkage analyses

The Zhengdan 958 RIL population along with the two parents were genotyped using a Maize SNP50 BeadChip (Ganal et al., 2011). Ten seeds from each genotype were germinated, and then young leaves were used for DNA extraction. The DNA quality was checked before genotyping. SNP genotyping was performed using GoldenGate assays (Illumina, San Diego, CA, USA) according to the manufacturer's protocol. SNP allele clustering and genotype calling was performed using Genome Studio v. 2011.1 software (Illumina). A genetic linkage map was constructed using MSTMap software (Wu et al., 2008).

### QTL analysis

For each Z_s_ (s = 1, 2) population, the trait averaged values of three replicates for each environment were used for QTL analysis. The adjusted mean (BLUP) values for each trait across five environments were used for a combined analysis. Composite interval mapping (CIM) implemented in Windows QTL Cartographer version 2.5 (Zeng, 1994; Wang et al., 2006) was employed. Model 6 from Zmapqtl was used, and 5 markers were set up as the number of cofactors. The QTLs were scanned with a 0.5-cM interval between markers. Permutation tests with a minimum of 1,000 replicates were adopted to determine the thresholds for the logarithm of odds (LOD) scores of putative QTL (Churchill and Doerge, 1994). The mapped QTL in Z_1_ and Z_2_ reflect the augmented additive effects $a_{i}^{*}$ and augmented dominance effects $d_{i}^{*}$, separately (Melchinger et al., 2007). According to the scale of dominance degree commonly used in previous studies (Stuber et al., 1987; Jiang et al., 2015), the dominance degree ratios were estimated as |$d_{i}^{*}$/$a_{i}^{*}$| = augmented dominance effects/augmented additive effects: A, additive (|$d_{i}^{*}$/$a_{i}^{*}$| ≤ 0.20); PD, partial dominance (0.20 < | *d*_i_*/*a*_i_*| < 0.80); D, dominance (0.80 ≤ |$d_{i}^{*}$/$a_{i}^{*}$| < 1.20); and OD, overdominance (|$d_{i}^{*}$/$a_{i}^{*}$| ≥ 1.20). QTL confidence intervals were determined based on positions ±2 LOD away from the peaks of the likelihood ratios (LRs) (Zhai et al., 2016). QTLs with overlapping confidence intervals were treated as congruent. Based on the mixed model approach described by Wang et al. (1999), digenic epistasis QTL was analyzed using QTLMapper. The epistatic effects observed in Z_1_ and Z_2_ stood for additive by additive (AA) and dominance by dominance (DD) interaction effects, respectively.

## Results

### Heterosis and population performance

The average field performances of eight EW-related traits for the hybrid Zhengdan 958 and its parental lines (Zheng 58 and Chang 7–2) for five environments are listed in Table 1. Zheng 58 had higher RSN and HSW compared to Chang 7–2, whereas ERN, ESN, and ED of Chang 7–2 were significantly higher than those of Zheng 58 (*P* < 0.01). Compared to parental lines, hybrid Zhengdan 958 exhibited overwhelming superiority in all eight traits. Notably, EW and ESW showed high MPH (138.61 and 152.87%), followed by ESN, RSN, and EL (67.21, 56.25, and 39.60%), and ED and ERN were relatively smaller (22.09 and 16.80%).

**Table 1:** **Performance of the basic generations (the parental line Zheng 58, Chang 7–2, and the hybrid Zhengdan 958) and heterosis for eight EW-related traits**.

**Generation**	**ERN**	**ED (cm)**	**RSN**	**EL (cm)**	**HSW (g)**	**ESN**	**ESW (g)**	**EW (g)**
Zheng 58	12.94 ± 0.61	3.99 ± 0.18	28.18 ± 2.17	16.29 ± 1.05	29.98 ± 1.89	367.29 ± 26.87	92.88 ± 8.23	105.57 ± 11.09
Chang 7–2^a^	14.28 ± 1.63^**^	4.42 ± 0.10^**^	26.80 ± 2.22^*^	12.05 ± 0.97^**^	24.55 ± 1.23^**^	406.92 ± 32.53^**^	95.75 ± 7.95	108.05 ± 7.74
MP	13.61 ± 0.80	4.21 ± 0.084	27.49 ± 1.10	14.17 ± 0.71	27.20 ± 1.11	387.11 ± 22.66	94.31 ± 5.16	106.81 ± 6.62
F_1_^b^	15.89 ± 0.82^**^	5.13 ± 0.12^**^	42.93 ± 1.90^**^	19.80 ± 1.06^**^	34.71 ± 1.47^**^	617.76 ± 47.98^**^	236.82 ± 17.05^**^	254.14 ± 14.86^**^
MPH(%)	16.80 ± 3.42	22.09 ± 3.79	56.25 ± 6.42	39.60 ± 3.37	28.05 ± 3.86	67.21 ± 8.32	152.87 ± 22.03	138.61 ± 17.55

* P ≤ 0.05;

** *P ≤ 0.01*.

a Comparison between Zheng 58 and Chang 7–2 using t-test;

b *Comparison between MP and F_1_ using t-test*.

Mean values and broad sense heritability ($h_{B}^{2}$) of Z_1_ and Z_2_ for each trait are listed in Table 2. The $h_{B}^{2}$ in the Z_1_ population ranged from 0.80 to 0.96, among which ERN, RSN, EL, HSW, and ESN had high heritability ($h_{B}^{2}$ > 0.90). For the Z_2_ population, $h_{B}^{2}$ varied from 0.71 to 0.91. Remarkably, the $h_{B}^{2}$ of ESW and EW in Z_2_ was higher than in Z_1_, whereas the rest were lower compared to Z_1_.

**Table 2:** Phenotypic means, ***V***_***A***_, ***V***_***D***_, broad sense heritability ($h_{B}^{2}$), and average degree of dominance (D^*^) for Z_**1**_ and Z_**2**_ across different environments.

**Linear transformations**	**Statistic**	**Traits**							
		**ERN**	**ED**	**RSN**	**EL**	**HSW**	**ESN**	**ESW**	**EW**
Z_1_	Mean ±*SD*	14.83 ± 0.75	4.60 ± 0.09	36.35 ± 1.9	16.83 ± 1.01	31.40 ± 1.67	539.58 ± 35.08	160.98 ± 10.42	181.36 ± 11.61
	*V_*A*_*^*a*^	8.83^**^	0.20^**^	65.20^**^	17.32^**^	48.26^**^	22654.80^**^	2243.95^**^	2942.37^**^
	$h_{B}^{2}$	0.96	0.80	0.91	0.92	0.92	0.92	0.86	0.87
	CI ($h_{B}^{2}$)^*c*^	(0.95, 0.97)	(0.76, 0.84)	(0.89, 0.92)	(0.90, 0.94)	(0.90, 0.93)	(0.90, 0.93)	(0.83, 0.89)	(0.84, 0.89)
Z_2_	Mean ±*SD*	–1.02 ± 0.52	–0.11 ± 0.13	–1.16 ± 3.05	1.42 ± 1.18	3.15 ± 1.88	–52.86 ± 52.02	–6.47 ± 24.67	–3.38 ± 27.72
	*V_*D*_*^*b*^	6.14^**^	0.45^**^	185.66^**^	27.87^**^	75.23^**^	57830.42^**^	11632.47^**^	14007.08^**^
	$h_{B}^{2}$	0.79	0.71	0.89	0.86	0.84	0.89	0.91	0.91
	CI ($h_{B}^{2}$)	(0.75, 0.83)	(0.64, 0.76)	(0.87, 0.91)	(0.83, 0.89)	(0.81, 0.87)	(0.87, 0.92)	(0.89, 0.93)	(0.89, 0.93)
	D^*^	0.59	1.06	1.19	0.90	0.88	1.13	1.61	1.54

** *P ≤ 0.01*.

a Additive variance;

b Dominance varianc;

c *95% confidence interval*.

Variance analysis of Z_1_ and Z_2_ showed that *V*_*A*_ and *V*_*D*_ for all traits were significant (*P* < 0.01) (Table 2). Furthermore, we calculated the average degree of dominance (D^*^) for each trait. The results revealed that the D^*^ was > 1 for RSN, ESN, ESW, and EW and was < 1 for ERN, EL, and HSW.

Correlation coefficients among the eight traits within Z_1_and Z_2_ are listed in Table 3. Significantly positive correlations were observed between EW and six traits for both Z_1_ and Z_2_, including HSW, RSN, ED, EL, ESN, and ESW. Notably, HSW had positive correlations with ED and EL and ESW but negative correlations with ERN and ESN in Z_1_. In addition, EL was positively correlated with ED and ERN in Z_2_ but was negatively correlated in Z_1_.

**Table 3:** **Correlation analysis within Z_**1**_ and Z_**2**_ for EW-related traits**.

	**RSN**	**ED**	**ERN**	**ESN**	**ESW**	**EL**	**EW**
HSW	−0.04 NS^a^	0.22^**^	−0.54^**^	−0.42^**^	0.42^**^	0.22^**^	0.39^**^
	0.52^**^^b^	0.56^**^	−0.04 NS	0.41^**^	0.73^**^	0.58^**^	0.74^**^
RSN		−0.10 NS	−0.12 NS	0.70^**^	0.68^**^	0.85^**^	0.67^**^
		0.56^**^	0.26^**^	0.93^**^	0.89^**^	0.91^**^	0.89^**^
ED			0.58^**^	0.40^**^	0.49^**^	−0.05 NS	0.48^**^
			0.60^**^	0.70^**^	0.77^**^	0.52^**^	0.79^**^
ERN				0.62^**^	0.09 NS	−0.20^*^	0.18 NS
				0.57^**^	0.37^**^	0.18 NS	0.38^**^
ESN					0.60^**^	0.52^**^	0.61^**^
					0.88^**^	0.83^**^	0.89^**^
ESW						0.69^**^	0.96^**^
						0.86^**^	0.99^**^
EL							0.68^**^
							0.86^**^

* P ≤ 0.05;

** *P ≤ 0.01; NS, not significant*.

a Correlation in linear transformations Z_1_;

b *Correlation in linear transformation Z_2_*.

### Construction of the SNP-based genetic linkage map

In total, 905 SNP markers exhibiting polymorphisms between Zheng 58 and Chang 7–2 were adopted to construct the genetic linkage map (Appendix A). Of the 905 markers, 214 (23.6%) showed distortion segregation at *P* < 0.05, and 127 (14.0%) showed distortion segregation at *P* < 0.01. However, a previous study concluded that distorted markers will not just have a great effect on QTL detection (Zhang et al., 2010). As a result, these SNP markers were assigned to 10 chromosomes, spanning 2402.0 cM, with an average of 2.65 cM between adjacent markers (Figure S2 and Table S2).

### Mapping environmentally stable QTLs for Z_1_ and Z_2_

**Figure 1:**
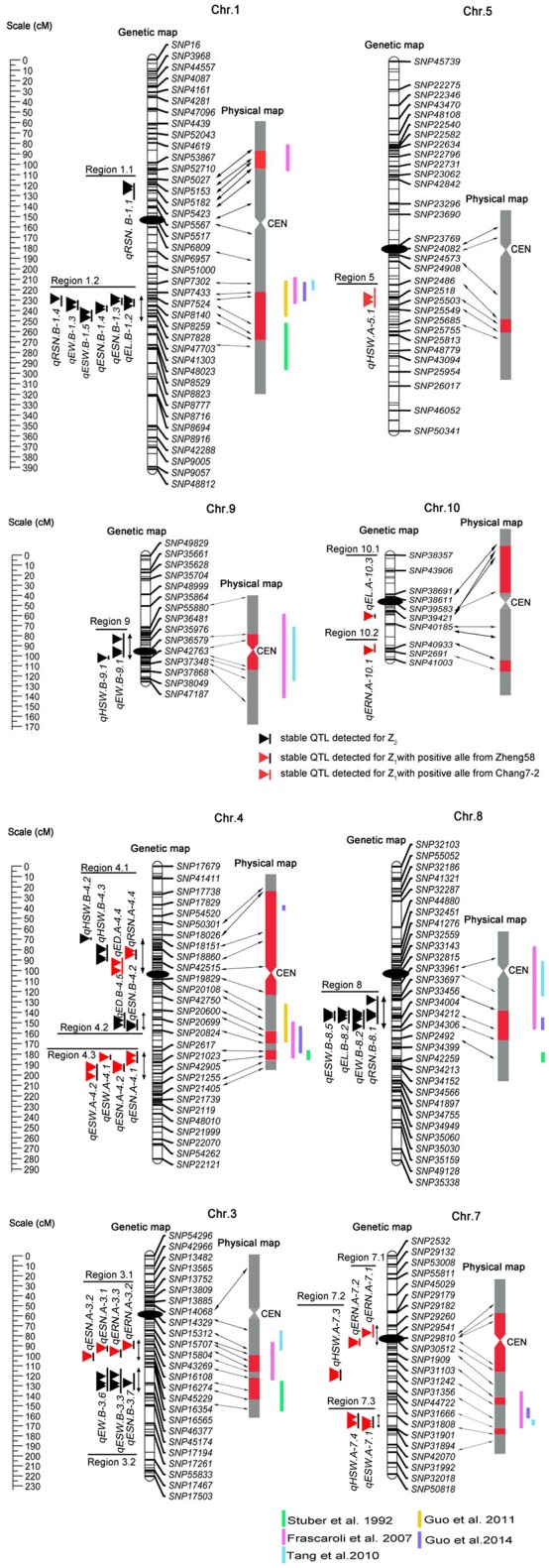
**Genetic locations of the 38 environmentally stable QTLs for EW-related traits**. The centiMorgan (cM) scale is shown on the left. Black ellipses indicate the approximate positions of the centromeres. Vertical bars in black or red represent the confidence interval of each QTL. A black vertical bar with black triangle represents heterotic-related QTLs detected for Z_2_; a black vertical bar with a red triangle represents additive QTLs with positive alleles from parent Zheng 58; a red vertical bar with a red triangle represents additive QTLs with positive alleles from parent Chang 7–2. Double-headed arrows represent the genomic regions characterized by QTL clusters. Red shadows on the physical map indicate the corresponding positions of each QTL. The verticals in different colors alongside the physical map indicate known heterotic-related QTLs from different studies (Stuber et al., 1992; Frascaroli et al., 2007; Tang et al., 2010; Guo et al., 2011, 2014).

Based on a genetic linkage map of 905 SNP markers, 483 QTLs were detected for eight traits for the Z_1_ and Z_2_ populations, which were distributed on all 10 chromosomes (Appendix B). We defined a QTL detected within two or more environments and in the combined analysis as an “environmentally stable QTL.” According to this criterion, 38 environmentally stable QTLs were detected on chromosomes 1, 3, 4, 5, 7, 8, 9, and 10 (Figure 1 and Table 4).

**Table 4:** **Genomic regions harboring environmentally stable QTLs for EW-related traits for Z_**1**_ and Z_**2**_**.

**Genomic regions^a^**	**Interval (cM)**	**Associated traits^b^**	**Included QTL**	**Z_1_^c^**			**Z**_**2**_			**Gene action^d^**	**Detected environment^e^**	**References^f^**
				**LOD**	a_i_*	***R^2^*(%)**	**LOD**	d_i_*	***R^2^*(%)**			
Region 1.1	117.8–122.1	RSN	*qRSN.B-1.1*				3.7	0.7	5.5	OD	E1,E5,C	2
**Region 1.2**	227.8–250.7	RSN	*qRSN.B-1.4*				5.0	0.9	8.7	OD	E3,E4,C	1,2,3,4,5
		EL	*qEL.B-1.2*				6.8	0.4	10.5	OD	E3,E4,C	
		ESN	*qESN.B-1.3*				10.0	21.5	16.8	OD	E3,E4,E5,C	
		ESN	*qESN.B-1.4*				8.6	20.9	15.6	OD	E2,E3,C	
		ESW	*qESW.B-1.5*				9.3	9.2	13.7	OD	E1,E3,E4,C	
		EW	*qEW.B-1.3*				8.8	10.5	14.0	OD	E3,E4,C	
**Region 3.1**	85.4–101.2	ERN (+)	*qERN.A-3.2*	6.2	0.2	9.8				A	E1,E2,E3,E4,E5,C	2,3
		ERN (+)	*qERN.A-3.3*	5.4	0.2	9.0				A	E1,E3,E4,C	
		ESN (+)	*qESN.A-3.1*	6.6	12.8	12.8				A	E2,E5,C	
		ESN (+)	*qESN.A-3.2*	7.5	14.1	15.0				A	E1,E2,E5,C	
**Region 3.2**	113.0–133.9	ESN	*qESN.B-3.7*				8.5	20.4	15.2	OD	E4,E5,C	1,2
		ESW	*qESW.B-3.3*				4.4	6.2	6.0	OD	E2,E4,E5,C	
		EW	*qEW.B-3.6*				4.2	6.9	6.0	OD	E4,E5,C	
**Region 4.1**	70.1–100.2	RSN (+)	*qRSN.A-4.4*	5.6	0.6	10.8				A	E1,E2,E3,C	5
		ED (–)	*qED.A-4.4*	4.2	−0.03	8.2				A	E2,E5,C	
		HSW	*qHSW.B-4.1*				3.4	0.5	5.4	OD	E1,E5,C	
		HSW	*qHSW.B-4.3*				7.1	0.6	10.7	OD	E1,E3,E4,E5,C	
**Region 4.2**	151.9–163.1	ESN	*qESN.B-4.2*				3.8	12.6	5.9	OD	E4,E5,C	2,4,5
		ED	*qED.B-4.5*				7.6	0.05	14.4	OD	E4,E5,C	
**Region 4.3**	187–203.9	ESN (+)	*qESN.A-4.1*	5.0	13.5	11.3				A	E2,E3,E4,C	1,2,5
		ESN (+)	*qESN.A-4.2*	6.7	15.9	13.8				A	E2,E3,C	
		ESW (+)	*qESW.A-4.1*	3.6	3.1	8.9				A	E2,E4,C	
		ESW (+)	*qESW.A-4.2*	4.3	3.1	8.9				A	E2,E4,E5,C	
Region 5	219.3–228	HSW (–)	*qHSW.A-5.1*	3.5	−0.4	5.3				A	E2,E4,C	
Region 7.1	69.4–90.3	ERN (–)	*qERN.A-7.1*	5.4	−0.2	9.2				A	E1,E2,E5,C	
		ERN (–)	*qERN.A-7.2*	4.8	−0.2	7.6				A	E1,E2,E5,C	
Region 7.2	114–124.6	HSW (+)	*qHSW.A-7.3*	4.7	0.5	7.6				A	E3,E5,C	
**Region 7.3**	161.2–173	HSW (+)	*qHSW.A-7.4*	11.0	0.8	20.6				A	E1,E3,E4,E5,C	2
		ESW (+)	*qESW.A-7.1*	4.3	3.1	8.1				A	E1,E4,C	
**Region 8**	134.3–150.9	RSN	*qRSN.B-8.1*				4.0	0.8	5.9	OD	E1,E2,E3,C	2,5
		EL	*qEL.B-8.2*				6.7	0.4	10.4	OD	E1,E5,C	
		ESW	*qESW.B-8.5*				7.1	10.8	9.5	OD	E1,E3,E5,C	
		EW	*qEW.B-8.2*				5.6	8.4	8.5	OD	E1,E3,E5,C	
**Region 9**	94.6–103.7	HSW	*qHSW.B-9.1*				7.1	0.8	10.6	OD	E1,E2,C	2,3
		EW	*qEW.B-9.1*				7.6	12.8	13.1	OD	E2,E3,E4,C	
Region 10.1	58.4–62.8	EL (+)	*qEL.A-10.3*	4.4	0.3	7.2				A	E3,E4,C	
Region 10.2	88.4–98.4	ERN (–)	*qERN.A-10.1*	6.7	−0.3	10.7				A	E1,E2,C	

a *The genomic regions shown in bold are the ones with pleiotropic effects*.

b *Traits are the ear row number (ERN), ear diameter (ED), number of seeds per row (RSN), ear length (EL), one hundred seed weight (HSW), ear seed number (ESN), ear seed weight (ESW), and ear weight (EW). The plus (“+”) and minus (“–”) signs within the brackets indicate Zheng 58 and Chang 7–2 contributed increasing alleles, respectively*.

c *QTL information for the combined analysis*.

d Degree of dominance: A, additive (|d_i_^*^/a_i_^*^| ≤ 0.20); PD, partial dominance (0.20 < | d_i_^*^/a_i_^*^| < 0.80); D, dominance (0.80 ≤ |d_i_^*^/a_i_^*^| < 1.20); and OD, overdominance (|d_i_^*^/a_i_^*^| ≥ 1.20).

e *C Indicates the combined QTL analysis based on the BLUP values across five environments*.

f *Heterosis-associated QTLs reported in previous studies: 1 Stuber et al. (1992); 2 Frascaroli et al. (2007); 3 Tang et al. (2010); 4 Guo et al. (2011); 5 Guo et al. (2014)*.

Forty-five QTLs associated with ERN were detected. Five environmentally stable QTLs for ERN were identified on chromosomes 3, 7, and 10, and they were designated *qERN.A-3.2, qERN.A-3.3, qERN.A-7.1, qERN.A-7.2*, and *qERN.A-10.1*. Parental line Zheng 58 contributed an increased additive effect (A) for *qERN.A-3.2* and *qERN.A-3.3*, which explained the 9.8 and 9.0% variation observed in the combined analysis. In contrast, parental line Chang 7–2 contributed increased additive effects (A) for *qERN.A-7.1, qERN.A-7.2*, and *qERN.A-10.1*, which explained 9.2, 7.0, and 10.7% of the variation in the combined analysis, respectively. No environmentally stable QTL was detected for Z_2_.

Sixty-four QTLs associated with ED were detected. Two environmentally stable QTLs were identified on chromosome 4 (*qED.A-4.4, qED.B-4.5*). Parental line Chang 7–2 contributed additive effects (A) for the increased ED of *qED.A-4.4*, which explained 8.2% of the ED variation for the combined analysis. *qED.B-4.5* showed an overdominance effect (OD) for Z_2_, which explained 14.4% of the variation for the combined analysis.

Fifty-six QTLs associated with RSN were detected. Four environmentally stable QTLs were identified on chromosomes 1, 4, and 8, which were designated *qRSN.B-1.1, qRSN.B-1.4, qRSN.A-4.4*, and *qRSN.B-8.1*, respectively. The parental line Zheng 58 contributed increased additive effects (A) for *qRSN.A-4.4*, which explained 10.8% of variation for the combined analysis. Notably, *qRSN.B-1.1, qRSN.B-1.4*, and *qRSN.B-8.1* showed an OD for Z_2_, which explained 5.5, 8.7, and 5.7% of variation for the combined analysis, respectively.

Of the 55 QTLs associated with EL, one environmentally stable QTL (*qEL.A-10.3*) on 10 with an additive effect (A) was detected. Two environmentally stable QTLs (*qEL.B-1.2, qEL.B-8.2*) exhibited an overdominance (OD) effect for Z_2_, which explained 10.5 and 10.4% of variation for the combined analysis, respectively.

Fifty-two QTLs were found to be associated significantly with HSW, six of which were environmentally stable and were found on chromosomes 4, 5, 7, and 9 (*qHSW.B-4.1, qHSW.B-4.3, qHSW.A-5.1, qHSW.A-7.3, qHSW.A-7.4*, and *qHSW.B-9.1*). Parental line Zheng 58 conferred an increased additive effect (A) for *qHSW.A-7.3* and *qHSW.A-7.4*, and each explained as much as 7.6 and 20.6% of the HSW variation for the combined analysis. The parental line Chang 7–2 conferred an increased additive effect (A) for *qHSW.A-5.1*, which explained 5.3% of the HSW variation for the combined analysis. Interestingly, *qHSW.B-4.1, qHSW.B-4.3*, and *qHSW.B-9.1* showed an OD for Z_2_, which explained 5.4, 10.7, and 10.6% of the variation for the combined analysis, respectively.

Seventy-seven QTLs were associated with ESN. Eight environmentally stable QTLs were mapped on chromosomes 1, 3, and 4 and were designated *qESN.B-1.3, qESN.B-1.4, qESN.A-3.1, qESN.A-3.2, qESN.B-3.7, qESN.A-4.1, qESN.A-4.2*, and *qESN.B-4.2*. Parental line Zheng 58 contributed increased effects for *qESN.A-3.1, qESN.A-3.2, qESN.A-4.1*, and *qESN.A-4.2* with additive effects (A) and explained 12.8, 15.0, 11.3, and 13.8% of the variation for the combined analysis. Remarkably, *qESN.B-1.3, qESN.B-1.4, qESN.B-3.7*, and *qESN.B-4.2* exhibited an overdominance (OD) effect for Z_2_ and explained 16.8, 15.6, 15.2, and 5.9% of the variation for the combined analysis, respectively.

Sixty-eight QTLs were found to be associated significantly with ESW, and six environmentally stable QTLs were detected on chromosomes 1, 3, 4, 7, and 8 (*qESW.B-1.5, qESW.B-3.3, qESW.A-4.1, qESW.A-4.2, qESW.A-7.1*, and *qESW.B-8.5*). Parental line Zheng 58 contributed additive effects (A) for the increased ESW of *qESW.A-4.1, qESW.A-4.2*, and *qESW.A-7.1*, which explained 8.9, 8.9, and 8.1% of the ESW variation for the combined analysis. *qESW.B-1.5, qESW.B-3.3*, and *qESW.B-8.5* showed an OD for Z2, and their explained variation for the combined analysis was 13.7, 6.0, and 9.5%, respectively.

Sixty-six QTLs associated with EW were identified. Four environmentally stable QTLs were detected on chromosome 1 (*qEW.B-1.3*), 3 (*qEW.B-3.6*), 8 (*qEW.B-8.2*), and 9 (*qEW.B-9.1*). Notably, these four environmentally stable QTLs detected for Z_2_ showed an OD, and each of them explained 14.0, 6.0, 8.5, and 13.1% of the EW variation for the combined analysis, respectively.

### Analysis of digenic interaction across the entire genome

Previous studies reported that epistasis also played a certain role in heterosis (Frascaroli et al., 2007; Jiang et al., 2015). In this study, we thus also analyzed digenic interaction. In total, 206 pairs of digenic epistatic QTLs were detected, and the numbers for Z_1_ and Z_2_ were 122 and 84, respectively (Appendix C). For each trait, both additive by additive (AA) and dominance by dominance digenic interactions were observed. The total variation explained by all digenic interactions either for Z_1_ or Z_2_, was < 25% for most traits. The highest values of accumulated *R*^2^ were found for HSW for Z_1_ (41.13%, E3) and for ERN for Z_2_ (35.97%, E1), respectively.

Interestingly, several digenic epistatic regions were mapped to the confidence intervals of QTLs (Appendix C). For example, the genetic region flanked by SNP18775 and SNP18742 on chromosome 4 was found to share an additive QTL for RSN via an AA interaction with the genetic region on chromosome 5. In addition, the genetic region flanked by SNP7302 and SNP7373 on chromosome 1 was found to interact with a genetic region flanked by SNP54321 and SNP25914 on chromosome 5 via a DD interaction, and both genetic regions shared QTL for HSW. However, it is necessary to note that most of digenic epistatic regions did not co-localize with the regions of main-effect QTLs.

## Discussion

### The role of allelic interactions in heterosis

Heterosis has a revolutionary influence on the maize breeding program. There has been considerable interest in the genetic basis of heterosis. Previous studies have detected multiple QTLs related to maize heterosis based on various experimental designs. However, the number and QTL positions were not always congruent among different crosses (Stuber et al., 1992; Lu et al., 2003; Frascaroli et al., 2007; Tang et al., 2010; Larièpe et al., 2012; Guo et al., 2014; Jiang et al., 2015). Our present study was conducted based on a genetic linkage map of 905 SNP markers in five environments, which can precisely define the positions and stability of QTLs. Of the 483 QTLs detected, thirty-eight environmentally stable QTLs for eight traits (ERN, ED, RSN, EL, HSW, ESN, ESW, and EW) were selected. To be specific, 18 of them were detected for Z_1_, and 20 were for Z_2_. Notably, all 20 environmentally stable QTLs detected for Z_2_ were characterized by positive OD. Interestingly, the heterosis level (MPH) and D^*^ of different traits were globally met with the proportion of “overdominant” QTL, which revealed a good consistency of classical genetic analysis and QTL analysis. For example, the traits with the highest heterosis level and average degree of dominance, particularly EW and ESW, were the ones that showed the highest proportion of the OD. In contrast, traits with the lowest heterosis level and average degree of dominance, such as ERN, were the ones that showed the lowest proportion of the OD. These results may suggest the distinct genetic architectures of studied traits. The phenomenon of the overwhelming superiority of OD was reported by several research groups (Frascaroli et al., 2007; Larièpe et al., 2012). However, pseudo-overdominance cannot be excluded. For example, Graham et al. (1997) dissected an overdominant QTL on chromosome 5 associated with grain yield into two linked dominant QTLs by fine mapping. Li et al. (2015) showed that two separate loci with a repulsion linkage could appear as a single locus with an overdominance mode of inheritance. Therefore, to uncover whether the overdominance effects found are true, fine-mapping strategies should be used in future work.

Comparison analysis revealed that the heterosis related genomic regions in this study were also reported for yield and/or yield-related traits in different studies (Figure 1 and Table 4). For instance, genomic region 1.2 was detected to have pleiotropic effects for EW, ESW, ESN, EL, and RSN in the present study and was reported to affect grain yield and the number of kernels per plant by Stuber et al. (1992) and Frascaroli et al. (2007, 2009) as well as by Larièpe et al. (2012) for grain yield. The genomic region 3.2 that we highlighted for ESN, ESW, and EW was determined to be a heterotic locus for plant height, number of kernels per plant, and grain yield by Frascaroli et al. (2007, 2009). Region 8 appeared to be involved in EW, ESW, RSN, and EL, and Frascaroli et al. (2007) reported that it was a QTL cluster for grain yield, 100-seed weight, number of kernels per plant and plant height, in addition to a report by Tang et al. (2010) for grain yield. Remarkably, we found that genomic regions for heterosis of yield or yield-related traits were not always congruent between any two studies, which suggested that the combination of heterotic loci in different hybrids was genotype-dependent (Figure 1 and Table 4). Collectively, it can be seen that QTL analysis of different hybrids would broaden our understanding of the mechanism of heterosis. Moreover, these data provided further evidence for the notion that potential utilization of QTLs for heterosis is feasible by pyramiding if we treat them as inherited units, which deserves further investigation (Lu et al., 2003).

### Epistasis effect on heterosis

Epistasis, which is an important genetic phenomenon of interactions between non-allelic genes, has been reported to exert certain roles in heterosis in maize (Frascaroli et al., 2007; Ma et al., 2007; Tang et al., 2010). In this study, the number of epistatic interactions in a single environment ranged from 0 to 5 for a given trait. The degree of epistatic interactions varied in different environments but at a relatively low level with *R*^2^ < 25% for total epistatic interactions, either for AA or DD. The low level of epistasis effects was documented in the literature. For example, Ma et al. (2007) reported that 44 pairs of interactions for yield and its components were found and that the total contribution of the digenic interactions was 7%. Notably, only a few cases involved loci in epistasis interactions that were co-localized with main effect QTLs, as confirmed in previous studies (Frascaroli et al., 2007; Ma et al., 2007; Zhang et al., 2014). One of the causal reasons may result from the limitation of Design III in separating the QTL effect and their epistatic interactions with other QTLs in the analysis of heterosis (Melchinger et al., 2008). Additionally, no environmentally stable epistasis interaction was detected for any trait, which suggested that epistatic interactions were susceptible to environmental influence.

### Pleiotropic of environmentally stable QTLs

Because multiplication effects are a main cause of heterosis for grain yield in maize, it was difficult to dissect the genetic components underlying this complex trait. In this study, we chose EW and its secondary component traits for detailed analysis because these traits were less complex and because the results may be more credible and intuitive (Williams, 1959; Hua et al., 2003). Our data revealed that significant positive correlations were observed between EW and EW-related traits (Table 3). Theoretically, QTLs for EW and its components would be overlapped to some degree. As expected, a total of 10 genomic regions contained 33 co-localized environmentally stable QTLs, among which region 7.1 contained two tightly linked QTLs for same trait (ERN), whereas the other 9 genomic regions harbored QTLs for two or more different traits (Figure 1 and Table 4). For example, genomic region 1.2 affected five traits (EW, ESW, ESN, EL, and RSN) simultaneously, and all of them showed an overdominance effect OD. Region 8 harbored four QTLs for RSN, EL, ESW, and EW, with OD, and regions 3.2, 4.2, and 9 harbored overdominant QTLs for 3, 2, and 2 traits, respectively. Interestingly, region 4.1 contained two tightly linked overdominant QTLs for HSW and two additive QTLs for RSN and ED. In addition, additive QTLs harbored in genomic regions 3.1, 4.3, and 7.3 contributed to two EW-related traits, which agreed with the significant correlation between them. However, similar to the case of previous studies (Kusterer et al., 2007; Larièpe et al., 2012), we cannot determine that QTLs in the same genomic region were pleiotropic and/or closely linked QTLs for the reason of limited samples and relatively large confidence intervals for QTL positions.

### The usefulness of Design III in dissecting the genetic basis of heterosis

In this study, we adopted a variant of Design III from the single cross between maize inbred lines Zheng 58 and Chang 7–2 to analyze QTLs contributing to heterosis. The detection by the modified Design III can not only determine the precise locations of heterotic QTL but also estimate the augmented QTL effect $a_{i}^{*}$ and $d_{i}^{*}$ (Melchinger et al., 2007). The Design III population also possessed several advantages. First, the whole population could be recreated, which would allow experiments with replications under many different environments to be conducted, and this population could even be used for alternate experimental schemes. Second, similar to immortalized F_2_ populations, the phenotypic values used to evaluate heterosis come from hybrids instead of progenies, which may lead to inbreeding depression (Hua et al., 2003). Third, as shown in previous studies, this approach offers opportunities for analyzing heterosis (Hua et al., 2003; Frascaroli et al., 2007; Jiang et al., 2015). Recently, Guo et al. (2011) evaluated a set of 231 F_2:3_ families derived from the same hybrid Zhengdan 958 at two different plant densities to analyze the genetic basis of 12 yield-related traits. A comparison analysis revealed that two heterotic QTL regions were detected in both studies, including region 1.2 and 4.2 (Figure 1 and Table 4). For example, the ones on chromosome 1.08 for Ear weight, Grain weight per ear, Ear length, 100-kernel weight, and Kernel number per row was related to EW, ESW, ESN, EL, and RSN in our study. Nevertheless, the heterotic QTLs with an OD in regions 1.1, 3.2, 4.1, and 9 in our study were not reported by Guo et al. (2011). Collectively, our data showed that Design III was more powerful for analyzing heterotic QTLs than F_2:3_ families.

## Author contributions

YZ conceived the project; QY developed the Design III population; HL, MZ carried out experiments; HL, LG analyzed experimental results; HL, ZN, and YZ wrote the manuscript.

### Conflict of interest statement

The authors declare that the research was conducted in the absence of any commercial or financial relationships that could be construed as a potential conflict of interest.

## Abbreviations

QTL: Quantitative trait locus
RIL: Recombinant inbred line
TC: Testcross
ERN: Ear row number
ED: Ear diameter
RSN: Number of seeds per row
EL: Ear length
HSW: One hundred seed weight
ESN: Ear seed number
ESW: Ear seed weight
EW: Ear weight
MPH: Mid-parent heterosis
*D*^*^: Average degree of dominance
AA: Additive by additive interaction effects
DD: Dominance by dominance interaction effects.
