# Supplementary material for: Identification of Heterosis-Associated Stable QTLs for Ear-Weight-Related Traits in an Elite Maize Hybrid Zhengdan 958 by Design III
Source: Front Plant Sci. 2017 Apr 19;8:561. doi: 10.3389/fpls.2017.00561 (PMC5395643; doi:10.3389/fpls.2017.00561)
Supplement: Supplementary file 2 [file DataSheet2.docx]

**SUPPLEMENTARY MATERIAL**

**Table S1 |** Environments in which the TCs, RILs and the basic generations (the parental line Zheng 58, Chang 7-2 and the Hybrid Zhengdan 958) were evaluated

**Table S2 |** The distribution and density of SNP markers on the 10 maize chromosomes

**Figure S1 |** Experimental sites in five environments. The three locations selected for evaluation of TC populations along with other materials (i.e., the parental lines, F_1_ and RILs) are representative of three crucial maize-production zones in China: North China spring maize area (1); Huanghuaihai plain summer maize area (2); Northwest China maize area (5).The rest maize production zones are: Southwest China maize area (3); South China maize area (4); Tibet maize area (6) (Qiu et al. 2003; Xiong et al. 2007).

**Figure S2 |** Genetic linkage map generated from 174 individuals. The numbers beside the left bar indicate the genetic position of markers; and the numbers on the bottom indicate the chromosomes.

**Appendix A |** Genotypic data of the Zhengdan 958 RIL population that used in QTL analysis.

**Appendix B |** QTLs detected for EW-related traits in Z_1_ and Z_2_.

**Appendix C |** Epistatic QTLs detected for EW-related traits in Z_1_ and Z_2._

Table S1 Environments in which the TCs, RILs and the basic generations (the parental line Zheng 58, Chang 7-2 and the hybrid Zhengdan 958) were evaluated

| Environment | Location | Growing season | Latitude, | Annual rainfall （mm） | Average daily temperature (°C) |
| --- | --- | --- | --- | --- | --- |
|  |  |  | longitude |  |  |
| E1 | Gongzhuling, Jilin | 2012 | 124°81' E, 43°51' N | 705.50 | 6.08 |
| E2 | Jinghai, Tianjin | 2012 | 116°92' E, 39°03' N | 861.50 | 7.43 |
| E3 | Wulumuqi, Xinjiang | 2012 | 87°36' E, 43°46' N | 272.02 | 7.43 |
| E4 | Wulumuqi, Xinjiang | 2013 | 87°36' E, 43°46' N | 300.90 | 8.80 |
| E5 | Gongzhuling, Jilin | 2013 | 124°81' E, 43°51' N | 764.10 | 6.57 |

Table S2 The distribution and density of SNP markers on the 10 maize chromosomes

| Chromosome | Marker number | Genetic distance (cM) | Average genetic distance (cM) | Maximum genetic distance (cM) |
| --- | --- | --- | --- | --- |
| 1 | 130 | 392.07 | 3.02 | 28.11 |
| 2 | 116 | 271.62 | 2.34 | 13.19 |
| 3 | 80 | 218.76 | 2.73 | 16.86 |
| 4 | 129 | 290.49 | 2.25 | 13.41 |
| 5 | 102 | 355.33 | 3.48 | 16.08 |
| 6 | 61 | 138.82 | 2.28 | 11.78 |
| 7 | 62 | 218.05 | 3.52 | 16.57 |
| 8 | 124 | 281.21 | 2.27 | 24.96 |
| 9 | 58 | 125.79 | 2.17 | 20.99 |
| 10 | 43 | 109.85 | 2.55 | 11.99 |
| Total | 905 | 2401.98 | 2.65 | 28.11 |


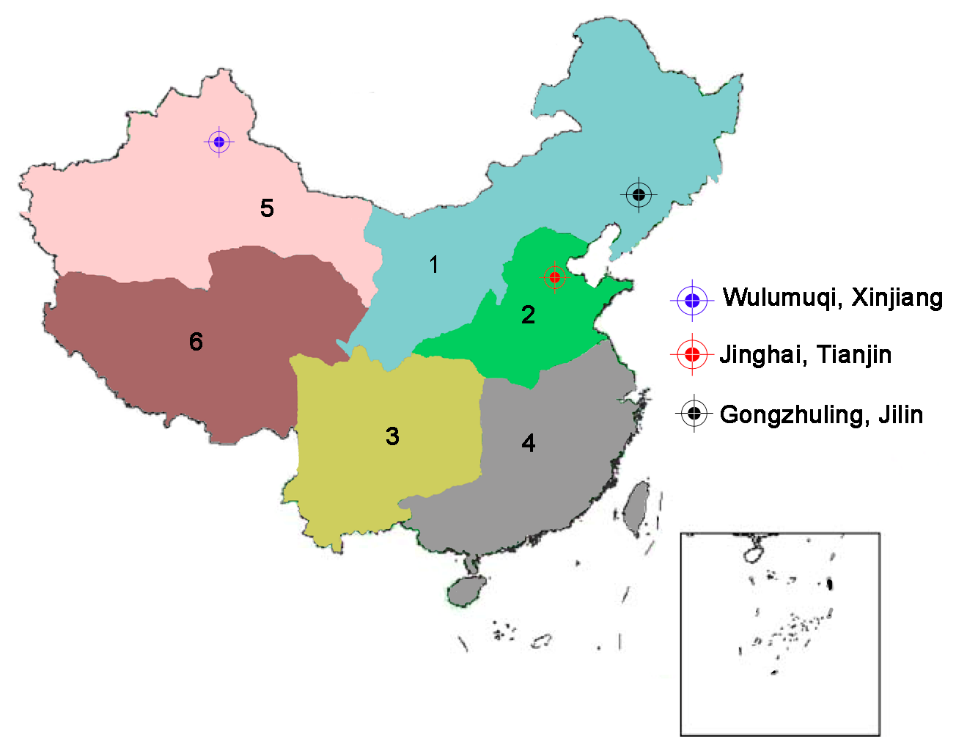


Figure S1 Experimental sites in five environments. The three locations selected for evaluation of TC populations along with other materials (i.e., the parental lines, F_1_ and RILs) are representative of three crucial maize-production zones in China: North China spring maize area (1); Huanghuaihai plain summer maize area (2); Northwest China maize area (5).The rest maize production zones are: Southwest China maize area (3); South China maize area (4); Tibet maize area (6) (Qiu et al. 2003; Xiong et al. 2007).

_
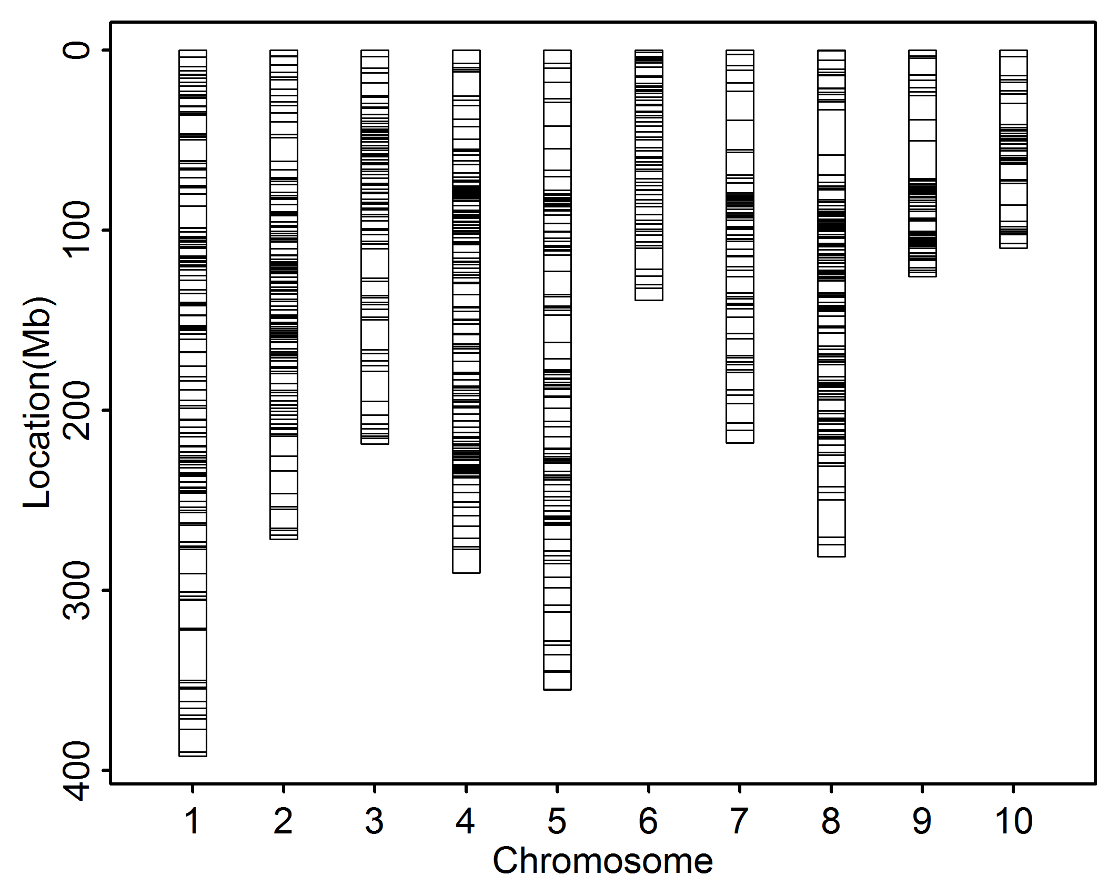
_

Figure S2 Genetic linkage map generated from 174 individuals. The numbers beside the left bar indicate the genetic position of markers; and the numbers on the bottom indicate the chromosomes.
